# Prosodic processing post traumatic brain injury - a systematic review

**DOI:** 10.1186/s13643-016-0385-3

**Published:** 2017-01-04

**Authors:** Gabriela Ilie, Michael D. Cusimano, Wenshan Li

**Affiliations:** 1 Faculty of Medicine, Dalhousie University, 5790 University Avenue, 4th Floor, Rm. 401, Halifax, NS B3H 4R2 Canada; 2 Dalla Lana School of Public Health and Department of Surgery, University of Toronto, Toronto, Canada; 3 Division of Neurosurgery, Keenan Research Centre and Li Ka Shing Knowledge Institute, St. Michael’s Hospital, Toronto, Canada; 4 Department of Psychology, University of Toronto, Toronto, Canada

**Keywords:** Traumatic brain injury, Prosodic processing, Speech prosody, Psychiatry, Recovery marker

## Abstract

**Background:**

Traumatic brain injury (TBI) survivors often report difficulties with understanding and producing paralinguistic cues, as well as understanding and producing basic communication tasks. However, a large range of communicative deficits in this population cannot be adequately explained by linguistic impairment. The review examines prosodic processing performance post-TBI, its relationship with injury severity, brain injury localization, recovery and co-occurring psychiatric or mental health issues post-TBI

**Methods:**

A systematic review using several databases including MEDLINE, EMBASE, Cochrane, LLBA (Linguistics and Language Behaviour Abstract) and Web of Science (January 1980 to May 2015), as well as a manual search of the cited references of the selected articles and the search cited features of PubMed was performed. The search was limited to comparative analyses between individuals who had a TBI and non-injured individuals (control). The review included studies assessing prosodic processing outcomes after TBI has been formally diagnosed. Articles that measured communication disorders, prosodic impairments, aphasia, and recognition of various aspects of prosody were included. Methods of summary included study characteristics, sample characteristics, demographics, auditory processing task, age at injury, brain localization of the injury, time elapsed since TBI, reports between TBI and mental health, socialization and employment difficulties. There were no limitations to the population size, age or gender. Results were reported according to the PRISMA guidelines. Two raters evaluated the quality of the articles in the search, extracted data using data abstraction forms and assessed the external and internal validity of the studies included using STROBE criteria. Agreement between the two raters was very high (Cohen’s kappa = .89, *P* < 0.001). Results are reported according to the PRISMA guidelines.

**Results:**

A systematic review of 5212 records between 1980 and 2015 revealed 206 potentially eligible studies and 8 case-control studies (3 perspective and 5 retrospective) met inclusion and exclusion criteria for content and quality. Performance on prosodic processing tasks was found to be impaired among all participants with a history of TBI (ages ranged from 8 to 70 years old), compared to those with no history of TBI, in all eight studies examined. Compared with controls, individuals with a history of TBI had statistically significantly slower reaction time in identifying emotions from prosody and impaired processing of prosodic information that is muffled, non-sense, competing, or in conflict (prosody versus semantics). Heterogeneous findings on correlations between specific brain locations and prosodic processing impairment were reported. Psychiatric issues, employment status or social integration post-TBI were scarcely reported but, when reported, they co-occurred with a history of TBI and prosodic impairments.

**Conclusions:**

The current review confirms the relationship between impaired prosodic processing and history of TBI. Future studies should collect and report comprehensive details about severity of TBI, location of brain injury and time elapsed since injury, as they could key influence factors to the extent of prosodic processing impairments and recovery from auditory processing impairments post-TBI. The exploration of prosodic processing tasks as a possible neuropsychological marker of TBI diagnosis and recovery is warranted.

## Background

Over the past 15 years, traumatic brain injuries (TBIs) have been on the rise and have become a public health care issue in North America [1–10]. The main mechanism of TBI among infants age 0–4, are falls, among adolescents age 10 to 20 are sport related injuries, and among adults are traffic collisions, falls, and assaults [2–4, 7, 11, 12]. Fall-related head injuries are approximately four times greater among older adults (over 65) compared to adults less than 65 years old [2–4]. Based on hospitalized and non-hospitalized records, estimates indicate that more males than females sustain TBIs [1–4, 11]. TBIs have large societal and economic toll [8, 13] and also affect the individual as TBI affects quality of life, including challenges to the individual’s ability to return to work or school and sustain relationships with family, friends, and community [2–4, 7].

Symptoms associated with TBI involve sensitivity to sounds (increased irritability with loud and/or high pitched sounds) along with various physiological, cognitive, and emotional symptoms including dizziness, headaches, sensitivity to light, mood changes, irritability, diminished focused attention, and slow reaction time [2, 4, 5, 7, 8, 11, 14]. Post-TBI symptoms are difficult to diagnose and prognosis as to whether the symptoms will persist or lead to adverse conditions is difficult [8, 11, 14]. Cognitive, functional, occupational and physical outcomes, as well as social reintegration post-TBI often present a challenge and are issues that have drawn much scientific attention in recent years. For example, adults with a history of TBI report increased current daily cigarette smoking, use of cannabis and non-medically prescribed opioids have significantly higher odds of being currently diagnosed with ADHD and report a greater number of road-related aggression and traffic collisions [12, 15]. Several population studies of adolescents and adults revealed that history of TBI is associated with increased symptoms of depression, anxiety, negative affect, and suicidal ideation [12, 16, 17, 18]. Furthermore, a systematic review by Rogers and Read [19] found that TBI patients are particularly at risk of developing major depression, generalized anxiety disorder and post-traumatic stress disorder years after TBI. Past research has also shown that, not only do injured individuals exhibit impaired employment and productivity post-TBI [20, 21] but they also show impaired executive functions, exhibit higher levels of depression and use maladaptive coping mechanisms such as escape-avoidance mechanisms [21]. Since prosodic perception and decoding of meaning from prosodic information has been shown to be key to well-being, effective social communication and emotional functioning [22], it may be hypothesized that challenges in emotional decoding of communication through sounds following a TBI may disrupt these mechanisms of emotional, adaptive and social functioning.

Speech prosody refers to the melodic aspect of speech. Its function, like that of melody in music, is to convey the emotional content [22, 23] and is a necessary feature in effective social communication [23, 24]. Emotion perception and induction through speech rely on prosody and the emotional code it shares with other prosodic domains (e.g., music and environmental sounds) [22, 23]. To date, no research has explored the diagnosis and assessment of recovery post-TBI using prosodic processing (e.g., music, environmental). Research evidence does exist, however, to suggest that auditory-related functions such prosody and paralinguistic cues (recognition of emotions conveyed through the melodic aspect of speech) are vital to social communication and are susceptible to impairment following TBI [22, 25, 26]. Patients with TBI have been shown to have difficulties not only with general linguistic and paralinguistic abilities, specifically in understanding and producing paralinguistic cues, but also with displaying problems in understanding and producing basic communication tasks [25, 26]. However, the results of these studies have been restricted by small number of participants. Individuals with TBI also very often show a large range of communicative deficits that cannot be adequately explained by linguistic impairment. For example, even though TBI patients may perform normally on standardized aphasia tests, their ability to manage communicative interactions in their daily lives is greatly impaired [14, 25–27]. This is not surprising, since communicative abilities rely on linguistic (not being able to understand what is implied as in comprehension of sarcastic utterances), extralinguistic (ability to communicate through gestures), paralinguistic (recognizing emotions conveyed through the prosody), context and conversational aspects of communication [24–26, 28]. For example, TBI patients have trouble ignoring literal meanings of an utterance in order to comprehend sarcasm as well as make indirect inferential requests by giving hints [29, 30]. Angeleri et al. [24] found that TBI patients performed worse than controls on tasks requiring comprehension and production of extralinguistic, paralinguistic, context and conversational communication. Their performance was also impaired in comprehension and production of pragmatic phenomena such as deceit and irony. Impairments in paralinguistic communication may be a causal factor for antisocial behaviour, poor social relationships and aggressive conduct behaviours often reported by patients with TBI [12, 15, 17, 18]. To our knowledge, to date, no systematic review has examined the association between prosodic processing and history of TBI.

This systematic review aims to fill this gap in the literature by examining this relationship between prosodic processing and TBI. The review examines whether prosodic processing impairments are related to history of TBI, TBI severity, brain injury location and recovery. This review also aims to identify whether any co-occurring psychiatric or mental health issues in addition to prosodic impairments post-TBI, in the studies identified, are also reported. Findings from this review will help ascertain if the literature warrants future exploration of the idea that prosodic processing could act as a neuropsychological marker of TBI to help diagnose and index TBI recovery. Implications for the future use of prosodic processing as a neuropsychological marker of TBI through psychoacoustic manipulations of sounds in all auditory domains are discussed.

## Methods

### Sources

A systematic search of English-language literature using MEDLINE, CINAHL, EMBASE, Cochrane, LLBA (Linguistics and Language Behaviour Abstract), Web of Science, Scopus and PsychINFO (January 1980 to May 2015) was performed along with a manual search of the cited references of the selected articles and the search cited features of PubMed. Appendix 1 lists the search strategy performed on MEDLINE as an example of the literature search performed in each database. The search was limited to comparative analyses between individuals who had a TBI and non-injured individuals (control). This study was not registered with PROSPERO.

The review includes studies assessing prosodic processing outcomes after the following procedures: *traumatic brain injury, subdural hematomas, cerebral aneurysms, craniotomy (for glioma and meningioma), craniotomy for subdural hematoma, burr hole(s) for subdural hematoma, cerebral aneurysm repair by craniotomy and endovascular technique, ventriculoperitoneal shunt insertion and revision, endoscopic third ventriculostomy, surgical treatment of epilepsy, temporal lobectomy, amygdalohippocampectomy, hemispherectomy, callosotomy and other procedure for seizures, or other neurosurgical cranial procedures for brain tumors, and epilepsy*.

Articles that discussed the following outcomes: communication disorders, prosodic impairments, aphasia, and recognition of various aspects of prosody, were included and were examined for assessments and reports of prosodic processing impairments. Methods of summary included study characteristics, sample characteristics, demographics, auditory processing task, age at injury, brain localization of the injury, time elapsed since TBI, reports between TBI and mental health, socialization and employment difficulties in studies assessing TBI and auditory processing evaluations. There were no limitations to the population size, age or gender.

We collected the electronic records in an Endnote data file. Titles and abstracts of the electronic search results were screened by one of the authors (WL) to identify the relevant studies. One of the authors (WL) and an undergraduate student (SW) independently evaluated the quality of the articles in the search and extracted data using data abstraction forms. The STROBE (Strengthening the Reporting of Observational Studies in Epidemiology) criteria for quality assessment were applied to evaluate each article on study quality and external and internal validity [31]. Agreement between the two raters was very high (Cohen’s kappa = .89, *P* < 0.001). Results are reported according to the PRISMA guidelines [32].

Information was extracted primarily from the “Results”, “Discussion” and “Methods” sections with some input from the “Background” section. Information that was extracted included study characteristics, participant characteristics, localization and mechanisms of brain injury, severity of TBI, time-elapsed since injury, methods and results pertaining to prosodic processing post-TBI, author’s interpretation of results and conclusions. Internal validity was evaluated by examining the study design (blinding, statistical tests, reliability, participant recruitment, validity and biases) and external validity was based on whether or not the sample was representative of the entire population. Please note that the localization of brain injuries was reported based on the damage to the brain, not of the skull and surrounding protective tissues. However, localization was reported if damage to the surrounding tissue damaged the brain.

## Results

**Fig. 1:**
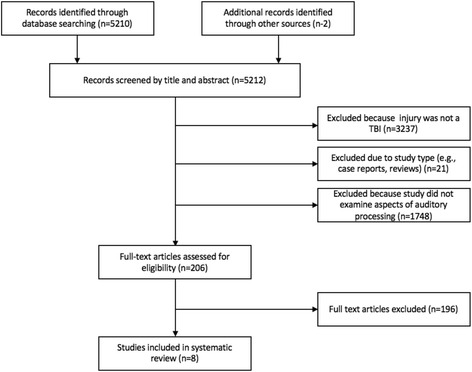
PRISMA diagram

Following the review of the databases searched, a total of 5212 records were obtained. Based on the inclusion and exclusion criteria, however, only 206 articles were retained for full-text examination as most articles did not report any assessment of prosodic processing; 8 were chosen to be included in this review. A PRISMA (Preferred Reporting Items for Systematic Reviews and Meta-Analyses) flowchart, shown in Fig. 1, was created to illustrate the number of articles found at each stage of data acquisition and the number of articles that were excluded at each stage. All included studies assessed various impairments in prosody processing. Table 1 displays the study characteristics. All studies were published after year 2000, with three conducted in the USA [33–35], two in the UK [36, 37], two in Australia [38, 39] and one in both, Canada and USA [40]. Three of the studies were prospective cohorts [33, 34, 40] while the others were retrospective. Four studies compared TBI prosodic performance with individuals who had orthopaedic injuries (control) [33, 34, 36, 40], while the others relied on healthy participants as controls. Sample sizes varied between 17 and 71 participants in each condition.

**Table 1:** Characteristics of the studies included in the review

Study	Study type	Journal published in	Study location	Exposure method—overall	Methods that assesses prosodic processing	Outcome measures	Limitations
Dennis et al. [40]	Retrospective cohort study	Journal of the International Neuropsychological Society	Canada and USA	Pictures of scenarios presented in forms of literal truth, ironic criticism and empathic praise as indicated by audiotapes of speaker’s utterances with neutral, ironic or empathic intonation. Participants were asked about facts and beliefs and to identify the intent of the speaker indicated by their tones	Participants must identify the intent of the speaker as indicated by their tones	Cognition, conation, identifying empathy and irony through prosody	While irony and empathy were presented through audio, the scenarios were delivered via pictures.
Dimoska et al. [38]	Retrospective cohort study	Journal of the International Neuropsychological Society	Australia	To assess participant's perception of emotion in voice, they completed two discrimination tasks using spoken sentences that varied in the amount of semantic information: that is, (1) well-formed English, (2) a nonsense language, and (3) low-pass filtered speech producing "muffled" voices. Participants also completed neuropsychological tests measuring impulsivity, cognitive/executive functions and inhibition of prepotent, automatic response	Materials: (1) audios of semantically well-formed or non-sense sentences with good phonetics and prosody spoken in various emotions and (2) muffled sentences without semantics and intact pitch/contour. Procedures: (1) same/different judgments for emotions portrayed by muffled and non-sense sentences and (2) identify emotional tone using prosody only or with semantics	Emotion recognition from voice	Small sample size, heterogeneous study population
Ietswaart et al. [36]	Prospective cohort study	Neuropsychologia	UK	(1) Labelling facial expressions and labelling morphed facial expressions, (2) labelling emotions from prosody and prosodic discrimination, (3) assessments for language comprehension deficits, (4) assessment of mental speed and pre-morbid intelligence and (5) test of depression and anxiety	(1) Emotional prosody discrimination: neutral sentences spoken in same/different emotional tone, (2) labelling emotions of neutral sentences spoken different emotional tones and (3) non-emotional prosody discrimination: sentences spoken in interrogative or declarative tone	Emotional recognition from face and prosody, depression and anxiety	Limited information for lesion analysis
McDonald and Saunders [39]	Retrospective cohort study	Journal of The International Neuropsychological Society	Australia	Ambiguous or neutral scenarios depicted in emotional (happy, surprised, angry, sad, fearful, disgusted) or neutral format. The emotional stimuli were presented via (1) audiovisual, (2) “still” photographs, (3) dynamic visual and (4) audio-only. Participants were presented with all four formats and asked to label emotions	Identifying emotions portrayed in audio and audio-visual format	Emotion recognition through various media formats	Controls had significantly more years of education than TBI
Milders et al. [37]	Retrospective cohort study	Journal of Clinical and Experimental Neuropsychology	UK	Participants were assessed for (1) emotional and behavioural consequences, (2) home integration, social integration and work integration (employment), (3) recognition of facial expression, (4) understanding intentions and social situation and (5) recognition of emotional prosody	Prosody discrimination: pairs of neutral sentences spoken in same/different (a) non-emotional tones and (b) emotional tones, (c) labelling emotional prosody and (d) labelling emotions when semantics conflicted with prosody	Emotional/behavioural issues, social integration, emotion recognition (face and prosody)	Small sample size, possible biased recruitment method
Schmidt et al. [33]	Prospective cohort study	Neuropsychologia	USA	Participants were assessed on recognition of emotions from voice and visual cues. They also did 2 control tasks, one that tests phonological discrimination and the other for face identity recognition	(1) Emotional prosody task: indicate emotions portrayed in audio of 4 semantically neutral sentences spoken in different emotional prosody and (2) phonological discrimination: match/non-match judgments made for 2 non-sense words that were identical or varied by a single phoneme	Labelling emotions from prosody and face, phonological discrimination	Only used one emotional prosody task, with limited trials
Schmidt et al. [34]	Prospective cohort study	Brain injury	USA	All were assessed on labelling emotional prosody, phonological discrimination and cognitive/neuropsychological tests at baseline and 3 months and underwent MRI at 3 months. DTI analysis was performed to investigate tracts that connect brain regions associated with emotional prosody	(1) Emotional prosody task: identify audio of neutral sentences spoken in different emotional prosody and (2) phonological discrimination: same/different judgments made for pairs of non-words that were identical or varied by a single phoneme	Emotional prosody recognition, phonological discrimination. Quantitative DTI variables	(1) Restricted to participants in a relatively acute stage of recovery and (2) did not take into account mechanism of injury
Zupan and Neumann [35]	Retrospective cohort study	Journal of Head Trauma Rehabilitation	USA	(1) Unimodal: facial affect recognition, (2) unimodal vocal affect recognition and (3) affect recognition from context-enriched multimodal medium. Participants were asked to identify emotions	(2) Vocal affect recognition (Diagnostic Analysis of Nonverbal Affect 2 -Voices): repetitions of neutral sentence spoken different emotional tones. Participants asked to indicate emotion portrayed	Recognition of emotions through voice, face and multimodal medium	No direct comparison for multimodal task (novel method); did not collect data on psychiatric issues

Table 2 displays the participant characteristics of each study. Three of the studies recruited children aged 8 to 17 while the rest recruited adults, usually with a wide age range of up to 70 years old [33, 34, 40]. Time elapsed between injury and assessment for four of the studies were within 5 years [33, 34, 36, 40], while other studies examined patients as far as 25 years post-injury or did not report specific duration. All eight studies reported the sex of the participants and overall had more male than female participants. Years of education were reported by five articles [35–39] and were matched between control and TBI participants. Severity of TBI was determined by Glasgow Coma Scale (GCS) scores [41], duration of post-traumatic amnesia (PTA), and loss of consciousness (LOC), sometimes also with confirmation of brain lesion from computed tomography (CT) scans. Specific criteria for determining mild, moderate, and severe TBI varied from study to study or were not reported. Mechanism of injury was also heterogeneous, including motor vehicle accidents (MVA), assaults, falls, blow to the head, bike-/sports-/recreation-related accidents, and work-related injuries [33, 36–40]. Finally, brain localization of injury was reported in five of the eight studies and included various brain areas [34, 36, 38–40].

**Table 2:** Characteristics of the population samples evaluated in the studies included in the review

Study	Study population	Characteristics matched	Age at assessment	Sex	Time between injury and assessment	Localization of brain injury, type of TBI/CHI	Mechanism of injury	Severity of TBI
Dennis et al. [40]	71 children previously hospitalized for TBI and 57 with orthopaedic injuries (OI) without loss of consciousness or brain injury. Exclusion: (1) history of serious injury, (2) premorbid neurological disorder or mental retardation, (3) child abuse or assault, (4) severe psychiatric disorder requiring hospitalization, (5) sensory or motor impairment that prevented assessment and (6) primary language other than English	Age at injury and assessment, sex, race, SES, mechanism of injury	TBI and OI controls: 8–13 years old	OI: 34 males, 23 females; TBI: 47 males, 24 females	12 and 63 months	(1) Focal lesion: mild/moderate TBI: 83%; severe TBI: 55%; (2) diffuse lesion: mild/moderate TBI: 50%; severe TBI: 45%; (3) skull fracture: mild/moderate TBI: 56%; severe TBI: 50%	(1) MVA: OI: 5%; mild/moderate TBI: 32%; severe TBI: 52%; (2) sports/bike/recreation: OI:72%; mild/moderate TBI: 38%; severe TBI:24%; (3) fall: OI: 23%; mild/moderate TBI: 30%; severe TBI: 5 24%	Mild/moderate: GCS scores 9–15 (*n* = 50); severe: GCS scores 3–8 (*n* = 21)
Dimoska et al. [38]	18 adults with moderate–severe TBI and 18 healthy controls. Inclusion: (1) experiencing social difficulties post-TBI, (2) sufficient cognitive and motor capacity to do study and (3) fluent in English. Exclusion: (1) premorbid neurological or psychiatric conditions, (2) current aphasia or agnosia and (3) current psychosis	Age, education	TBI: 22 to 63 years (mean 45.2; SD 11.7). Control: 23–62 years (mean 44.4; SD 12.1)	TBI: 13 males, 5 females	Mean time post-injury = 15.0 years (SD 9.5 months)	Mixed, as shown by CT scan	MVA: 11; assault: 4; fall: 2; blow to head: 1	PTA mean duration: 79.8 days (range 1–270 days); severity based on clinical judgment of CT scans/medical records and PTA, though specific criteria not specified
Ietswaart et al. [36]	30 TBI patients and 32 orthopaedic controls (OC). Inclusion for TBI: (1) diagnosis of TBI, (2) CT scan diagnosis or (3) evidence PTA. Exclusion criteria: (1) neurological or psychiatric history, (2) history of alcohol or drug dependency, (3) dementia or learning difficulties and (4) persistent post-injury language deficits; an extra exclusion criterion for OC was brain injury or PTA	Age, years of education, SES, sex	16–70 years old for TBI and OC	TBI: 25 males and 5 females. OC: 28 males and 4 females	Immediately after injury. Average interval = 2.1 (S.D. 1.8) months for TBI group. Follow-up: 1 year later	CT scans: 12 had damage mostly in frontal lobes, 6 mostly in temporal or parietal areas and 6 had diffuse lesions. No lesion information was available for six patients	Road traffic accidents: 13; fall from height: 5; other falls: 6; assault: 5; other causes: 3	Mild TBI: GCS 13–15 or PTA <24 h; moderate TBI: GCS 9–12 or PTA 1–7 days; severe TBI: GCS <9 or PTA >7 days
McDonald and Saunders [39]	34 adults with severe TBI and 28 healthy matched controls without neurological damage. Inclusion: (1) suffered a severe TBI resulting in altered consciousness of 1+ day and (2) fluent in English, not diagnosed with aphasia, normal sight and hearing. Mean length of PTA was 76 days (SD = 59)	Age, sex	TBI: 21 to 64 years (mean = 41). Controls: mean = 40.7 years, SD = 11.8	TBI: 9 females, 25 males. Controls: 22 males, 6 females	At least 1 year post-injury, on average 9.5 years post-injury (SD = 8)	Heterogeneous type (contusions, hemorrhages, hematoma, fracture) and location (bilateral, unilateral, frontal, parietal, temporal, occipital, basal ganglia) of injuries, with majority of frontal lobe lesions	MVA: 22; assault: 5; fall 5; work-related injury: 2	Mean length of PTA was 76 days (SD = 59). Specific criteria not reported
Milders et al. [37]	17 TBI patients without history of psychiatric disease or a premorbid alcohol or drug addiction. 17 healthy participants served as controls. A relative of each patient rated aspects of patient’s emotional and social behaviour before and after injury	Gender, age, years of education	TBI: 19 and 42 years (*M* = 30.5, SD = 13.3) Controls: *M* = 29.1, SD = 12.1	TBI: 7 females, 10 males. Controls: 7 females, 10 males	Mean = 4.4 years (SD = 4.9)	Not reported	Road traffic accident: 15; domestic accident: 1; assault: 1	Severe TBI: mean length of PTA = 33.6 days, SD = 27; GCS ≤8. Moderate TBI: GCS 9–12
Schmidt et al. [33]	69 children with orthopaedic injury (OI) and 75 with non-penetrating moderate to severe TBI. All were English speaking, had never previously been hospitalized for a head injury, were not injured by abuse and did not history of mental retardation or pervasive developmental disorder	Sex, SES	TBI and OI: 7–17 years of age at time of injury	TBI: 49 males, 26 females. OI: 50 males, 19 females	Assessed at 5 points in 2 years post-injury: baseline (within 1 month), then 3, 12, 18 and 24 months post-TBI	Not reported	7 motorcycle/moped; 5 bicycle; 12 fall; 4 sports/play; 12 hit by motor vehicle; 2 other	Moderate: lowest post-resuscitation GCS scores of 9–12 or GCS scores of 13–15 with brain lesions (contusions, hematomas) indicated by CT. Severe TBI: GCS scores of 3–8
Schmidt et al. [34]	45 children with moderate or severe TBI and 46 with orthopaedic injury (OI). Inclusion criteria: English speaking, no previous hospitalization for head injury, no previous diagnosis of a severe psychiatric disorder, mental retardation or a neurodevelopmental disorder	SES, sex	TBI and OI: 7–17 years at time of injury	TBI: 14 females; 31 males; OI: 13 females, 33 males	Immediately after injury and at 3 months	Heterogeneous, as demonstrated by MRI and tracked by DTI	Low speed = 29; high speed = 16	Moderate TBI: GCS score 9–12 or 13–15 with brain lesions (contusions, haematomas) indicated by CT scans. Severe TBI: GCS scores of 3–8
Zupan and Neumann [35]	60 adults with moderate to severe TBI and 60 healthy controls. TBI must have GCS score, PTA or LOC indicative of moderate/severe TBI. Exclusion: presence of developmental affective disorder, acquired neurological disorder, psychiatric disorder and/or impaired vision or hearing. Controls were excluded if they had history of TBI or concussion. For all, English was the primary language	Age	TBI: 21.6 to 63 years (mean = 40.98; SD = 12.45); control: 18 to 63.2 years (mean = 40.64; SD = 13.04)	TBI: 37 males and 23 females; control: 38 males and 22 females	At least 6 months post-injury	Not reported	Not reported	Moderate to severe TBI (GCS ≤12; PTA ≥24 h; LOC ≥24 h)

### Prosodic impairments post-TBI

Table 3 displays the results of the studies included in the review by pointing out associations between auditory processing outcome, localization of injury, TBI severity, age at time of injury, time elapsed since injury, social economic status (SES), executive function, mental health, socialization and employment difficulties. In all of the eight studies included in this review, prosodic impairments were the primary communication disorder and processing of prosody was the most examined aspect of prosodic processing. Seven of the eight studies reviewed assessed participants’ ability to label emotions through prosody as one of their primary outcomes [33–39]. Participants were asked to listen to semantically neutral sentences spoken in different emotional tones and identify the emotions portrayed. All seven studies found TBI patients to be impaired compared to established standard norms, healthy participants or OI controls [33–39]. Furthermore, Dimoska et al. [38] and Ietswaart et al. [36] found that reaction time for labelling emotional prosody was also longer for TBI participants. Five of the eight studies reviewed also examined facial emotion recognition in conjunction with prosodic emotion recognition [33, 35–37, 39]. Of these, four of the five found co-occurring prosody and facial emotion recognition impairments among TBI participants [33, 35–37], with the one exception being McDonalds and Saunders’ [39] study in which the authors did not find TBI participants to be impaired on facial emotion recognition. Little to no significant correlations were found between specific emotion labelling and poor prosodic decoding performance, nor did specific emotion labelling/mislabelling contribute to any group differences in performance. This suggests that prosodic impairments are not general and not specific to one particular labelling ability of basic emotions (e.g. happiness, fear, sadness).

**Table 3:** Evaluation of the results of studies included in the review by auditory processing outcome, localization of injury, TBI severity, age at time of injury, time elapsed since injury, social economic status (SES), executive function, mental health, socialization and employment difficulties

Study	Type of AP impairment	TBI impaired compared to controls (Yes/No)	Significant association with severity of TBI (Yes/No/Not assessed)	Significant association with location/type of brain lesion (Yes/No/Not assessed)	Significant association with age at injury (Yes/No/Not assessed)	Significant association with time elapsed since injury (Yes/No/Not assessed)	Significant association with SES (Yes/No/Not assessed)	Significant association with cognitive/executive functions (Yes/No/Not assessed)	Reports of mental health or socialization issues among the TBI group (Yes/No/Not assessed)	Impaired employment post-TBI (Yes/No/Not assessed)
Dennis et al. [40]	Prosody-identifying empathy and irony	Yes *η* ^2^ = .095	Yes: GCS score	Yes: focal CT abnormality score negatively associated with identifying literal truth, *B* = −3.96, SE = 1.74, *β* = −.29, *p* = .026, but not empathy and irony. No: diffuse CT abnormality score did not predict any outcome	Yes: older age positively correlated with better recognition of empathy, irony and literal truths; *η* ^2^ = .15	No	Not assessed	Not assessed	Not assessed	Not assessed
Dimoska et al. [38]	(1) Prosody—labelling emotions; (2) prosody: processing muffled or non-sense sentence	Yes: TBI, compared to controls, had overall greater difficulty with (1) and (2), reaction time was also slower	Not assessed	Those impaired on labelling had intracerebral and subdural hemorrhages, cerebral edema, or extensive injuries to left/right temporal lobes. Out of 4 participants with focal lesions in right frontal region, only 1 was impaired on labelling task	Not assessed	No	Not assessed	Yes: working memory (*r* = 0.520; *p* < .01) and verbal comprehension (*r* = 0.413; *p* < .05) was correlated with labelling emotions, but not for emotion discrimination	Yes	Yes
Ietswaart et al. [36]	(1) Prosody—labelling emotions, (2) prosody—discrimination of non-emotional tones, (3) prosody—discrimination of emotional tones	Yes: for (1) at initial assessment and follow up *η* ^2^ = .16; reaction time is also slower, *η* ^2^ = .21. No: for (2) and (3)	No	No	Not assessed	Yes: both TBI and orthopaedic injured controls improved at 1-year follow-up	Not assessed	Yes: correlation with verbal fluency, *r* = > .60, *p* = < .001. But impairment is still significant after controlling for cognitive abilities	Yes	Not assessed
McDonald and Saunders [39]	Prosody—labelling emotions	Yes: impaired for audio-visual and especially impaired for audio-only. Bonferroni adjusted confidence interval = 95%	Not assessed	No	Not assessed	Not assessed	Not assessed	Not assessed	Yes	Yes
Milders et al. [37]	(1) Prosody—labelling emotions, (2) prosody—discrimination of emotional tones and (3) non-emotional tones, *t*(32) = 2.6, *p* < .05, (4) prosody—semantics incongruent with prosody	Yes: for (1), (3) and (4). No: for (2)	Not assessed	Not assessed	Not assessed	Not assessed	Not assessed	Not assessed	Yes: TBI group was more impaired on pragnosia and suffered from depression compared to controls, behavioural and social problems were also elevated post-TBI	Employment significantly decreased after TBI. Pre-TBI: 13/17 patients were full- or part-time employed. Post-TBI: 5 were employed, and all 5 worked at lower level
Schmidt et al. [33]	(1) Prosody—labelling emotions, (2) phonological discrimination	Yes: impaired for all time points and for both (1) and (2)	Yes	Not assessed	Yes: younger age at injury associated with faster rate of recovery in emotional prosody performance	Yes	Yes: higher SES correlated with faster rate of improvement in emotional prosody performance	Not assessed	Not assessed	Not assessed
Schmidt et al. [34]	Prosody—labelling emotions; phonological discrimination	Yes: impaired for labelling emotions from prosody and correlated with performance on phonological discrimination	No	TBI had greater mean apparent diffusion co-efficient (ADC) values and lesser fractional anisotropy (FA) values. Within TBI, FA was related to Simple Emotion Score only in left cingulum bundle, *r* = −0.305, *p* = 0.047. FA of the genu of the corpus callosum was related to phonological discrimination, *r* = 0.407, *p* = 0.012	Not assessed	Not assessed	Yes: children with higher SES scores performed better than those with lower scores, *t*	Not assessed	Not assessed	Not assessed
Zupan and Neumann [35]	Prosody—labelling emotions	Yes, *η*_p_^2^ = 0.09	No	Not assessed	Not assessed	Not assessed	Not assessed	Not assessed	Not assessed	Not assessed

Among the reviewed articles, TBI participants were also assessed on their ability to label emotional prosody of muffled or non-sense sentences [38], identify emotional prosody irrespective of the semantics of the sentence [37], identify empathy and irony through prosody [40] and make same-different judgments of non-emotional (i.e. declarative or interrogative) [36, 37] and emotional prosodic tones of sentences [36, 37]. Impairments were evident for many of the assessments. Specifically, when asked to identify emotions conveyed through prosody in a context where the semantics of the sentence conflicted with the prosody of the utterance, TBI participants were more likely to choose the emotions conveyed through semantics instead [37]. Furthermore, TBI participants were not impaired when discriminating sentences spoken with the same or different emotional tones [36, 37], but showed impaired performance and slower reaction times when discriminating sentences spoken with the same or different non-emotional (declarative versus interrogative) prosodic tones [37]. An impairment in discriminating non-emotional prosody was, however, not observed by Ietswaart et al. [36] where impairments in labelling emotional prosody by TBI patients were observed when compared to controls.

Dimoska et al.’s [38] study examined performance on labelling emotional prosody of muffled or non-sense sentences and found it to be statistically significantly worse among TBI participants compared to healthy participants. In as separate study, performance on recognizing irony and empathy delivered through prosody was also worse among individuals with severe TBI compared to OI controls, although the TBI group performed equally well on identifying literal truths (in other words, when prosody converged with scenarios in pictures) compared with OI controls [40]. Participants with mild to moderate TBI were significantly worse than controls only for identifying irony, but not empathic praise. The authors noted that empathy might be easier to identify than irony because the intention of empathic prosody matches the semantics of the speech. Their findings point out that participants with TBI are particularly impaired in identifying intention through prosody.

### Phonological discrimination post-TBI

Phonological discrimination was assessed in two of the eight studies, by asking participants to make “matching” or “not-matching” judgments after two non-sense words that were either identical or varied by a single phoneme when presented [33, 34]. Since the primary goal of the two studies was to examine labelling emotions delivered through prosody, this task served as a control task for auditory processing issues that affect emotional labelling. TBI participants showed impaired performance on these non-prosodic processing tasks [33, 34]. Performance on phonological discrimination was also significantly and positively correlated with performance on labelling emotional prosody [33, 34], meaning that participants who performed worse on phonological discrimination also performed worse on labelling emotional prosody. The correlation was stronger for younger participants.

### Age at injury, time elapsed since injury and mechanism and location of injury

Since variables such as GCS, age at injury, time elapsed since injury and mechanism and location of injury are often heterogeneous, not reported, or not included in statistical analysis for the eight studies in this review, clear patterns of correlations between these variables and performance on prosodic processing tasks were not emergent. Studies examining the effects of severity of TBI on performance on prosody tasks found conflicting results. Schmidt et al. [33] found that GCS score was significantly correlated with performance on labelling emotional prosody. Participants with more severe forms of TBI performed worse than those whose injury was less severe on this task, and the correlation grew stronger over time. However, three studies did not find any correlations between GCS or PTA with performance on labelling emotional prosody [34–36]. For recognition of irony and empathy from prosody, Dennis et al. [40] found that GCS score was positively associated with recognizing empathy, but not irony or literal truths.

Results are also conflicting for effects of age at injury. Age at injury was found to be not correlated with performance on labelling [33, 35, 36] or discriminating [36] emotional prosody of neutral sentences. Schmidt et al. [38], however, found that younger age at injury was associated with a faster rate of recovery in emotional prosody performance for both TBI and OI controls groups. The relationship between performance on phonological discrimination tasks and on labelling emotional prosody tasks was also stronger for younger participants. Dennis et al. [40] also found that older age at injury was correlated with better performance on recognizing irony, empathy and literal truths from Time elapsed since injury appears to affect prosodic performance among TBI patients. Although studies that examined performance on prosody tasks only at one point in time generally found no significant correlations [35, 38, 40], studies that assessed participants at more than one time-point reported improvement in performance with the passage of time, although recovery was not complete. For example, Ietswaart et al. [36] found that performance for labelling emotional prosody was better for both TBI participants and OI controls at 1-year follow-up than immediately after injury, though TBI participants remained impaired compared to controls at both time points. Since both groups improved at follow-up, the improvement could be attributed to either recovery or familiarity with the tasks. A second study, by Schmidt et al. [33] also reported improvements in labelling emotional prosody throughout the five follow-up time points within 2 years post-injury among TBI participants compared to controls.

SES was often unreported or matched between TBI and control groups; its effects on prosodic processing was seldom reported. Two of the eight studies factored SES into their analyses and both found it to be a significant predictor for emotional prosody labelling performance [33, 34]. Schmidt et al. [34] found that TBI children from families of higher SES performed better on emotional prosody tasks than children from families of lower SES. Schmidt et al. [33] found that, though SES was not correlated with performance on labelling emotional prosody, it did affect how TBI and OI groups recovered. Among participants with lower SES, OI controls demonstrated a faster rate of recovery compared to TBI participants. For participants with higher SES, however, TBI participants demonstrated a faster rate of recovery than OI controls.

Location of brain injury was the variable that produced the most heterogeneous effects. Dennis et al. [40] found that focal CT abnormality score, though negatively associated with identifying literal truth, was not associated with identifying empathy or irony. Diffuse CT abnormality score did not predict any outcome. Ietswaart et al. [36] also found that frontal lobe damage, compared to damage in other areas, did not affect performance on labelling emotional prosody. Similarly, MacDonald and Saunders [39] found performance to be not related to laterality of injury or presence of anterior pathology. Meanwhile, results from Dimoska et al. [38] showed that participants impaired on labelling of emotional prosody were those with intracerebral and subdural hemorrhages, cerebral edema or extensive injuries to the left or right temporal lobes. In contrast, while four participants had focal lesions in the right frontal region, only one was impaired. Using diffusion tensor imaging (DTI), Schmidt et al. [34] also found that the TBI group had greater mean apparent diffusion co-efficient (ADC) values and lesser fractional anisotropy (FA) values than OI controls. Within the TBI group, FA was related to recognition of simple emotions through prosody only in the left cingulum bundle. FA of the genu of the corpus callosum was related to phonological discrimination. This is, however, a stark contrast to analysis within the OI group, which found many more correlations between performance on prosody and the various neuro-networks. Overall, results are too heterogeneous to point to any consistent significant correlations between impaired prosodic decoding impairment and a specific location of brain injury.

### Relationship between cognitive and executive function and prosodic processing post-TBI

Cognitive and executive functions were generally not significantly correlated with any emotional prosody tasks [37, 39, 40]. Iestwaart et al. [36] found a significant correlation between performance on labelling emotional prosody and verbal fluency, but impairment in prosody task remained significant for TBI survivors compared to controls after controlling for verbal and cognitive abilities. Only one study found that working memory and verbal comprehension were significantly correlated with labelling emotions, but the correlation was not present for discriminating emotional prosody [38].

Finally, psychiatric issues [37], employment [37, 39], or social integration post-TBI [37, 38] were also reported by some of the studies. Compared to controls, TBI participants reported more pragnosia and depression [37], behavioural problems [37, 38], impaired social integration [37, 38] and unemployment post-TBI [37, 39] than controls.

While prosodic and communication impairments are the primary language disorders discussed by the studies reviewed, three of the eight studies also assessed the presence of additional language impairments. Despite TBI participants clearly demonstrating communication difficulties as demonstrated by their prosodic impairments, these impairments were not captured by most standardized language tests. Specifically, Dimoska et al. [38] found that TBI participants did not score significantly lower on the Wechsler Test of Adult Reading (WTAR), a neuropsychological assessment tool that is highly correlated with measures of verbal IQ (*r* = .75) and verbal comprehension (*r* = .74) [42], than healthy controls. TBI participants in the study by Iestwaarts et al. [36] were impaired on the verbal alternating fluency test (switching between two semantic categories) and National Adult Reading Test (pronunciation of irregular words), but none scored below the cut-off point for the complex ideational subtest of the Boston Diagnostic Aphasia Examination which assesses for language comprehension deficits. Meanwhile, TBI patients were more impaired on functional communication—an integration of cognitive, linguistic and speech articulatory abilities that enable an individual to communicate effectively and appropriately in daily situations [43]. For example, Milders et al. [37] found that TBI participants had high pragnosia score, demonstrating deficits in pragmatics, understanding meaning through context of the utterances, intent of the speakers and other prosodic factors [24]. TBI participants in the study by Dimonska et al. [38] also reported having trouble understanding what people were saying, being inappropriate in conversations, having difficulties in understanding and producing humor and displaying rigidity in verbal communications.

## Discussion

The results of this review indicate that the link between language processing and TBI has been clearly understudied. Yet, all eight studies reviewed here found prosodic processing impairments among participants with a history of TBI, compared to controls. TBI participants showed impaired performance and slower reaction time in identifying emotions from prosody, as well as impaired processing of prosodic information that are muffled, non-sense, competing or in conflict (prosody versus semantics). These results are congruent with past studies examining prosodic processing of TBI patients [44–47]. Cockrell et al. [48] found that 16% of TBI children in their study had central prosodic processing problems. Bergemalm and Borg [49] also evaluated patients with closed-head injuries using peripheral and central prosodic tests as well as questionnaires about hearing ability and quality of life and found that 68% of patients demonstrated abnormalities on one or more audiometric tests. Fourteen of their 25 patients also scored lower on pure-tone audiometry and/or central audiometric tests, and many showed significant progressive deterioration. Taken together, these results illustrate the importance of assessing TBI participants on various aspects of prosodic processing both during the acute phase of the injury and in later follow-ups.

Interestingly, three of the studies reviewed that assessed, in addition to prosodic impairments and other communication difficulties, reading tests, found impairments in some prosodic processing outcomes and not others [36–38]. Specifically, while some participants were impaired on verbal fluency and National Reading Test for Adults (NART), others performed no different than controls on the Wechsler Test of Adult Reading (WTAR) and the Boston Diagnostic Aphasia test. Impairments in functional communication (which requires individuals to match linguistic acts with paralinguistic elements), however, were much more prominent among individuals with TBI compared with controls, showing TBI-related prosodic processing deficits. Such findings are not surprising. TBI causes a wide range of communication impairments (e.g. aphasia) that cannot be adequately diagnosed by standard language tests [50–52]. On the other hand, paralinguistic factors such as tone, intonation, rhythm and prosody are crucial in functional communication [53]. Findings showing that TBI patients exhibit prosodic impairments demonstrate that they are indeed struggling with prosodic processing of paralinguistic cues, which although may not be detected by standard language tests can impede effective communication.

Among all eight studies reviewed, prosodic impairments among TBI patients were demonstrated by impairments in identifying emotions and affective meaning communicated through prosody. Although when processing emotional meaning through auditory cues, both linguistic and prosodic information are used, prosody alone becomes extremely important for accuracy in decoding under conditions of ambiguity, sarcasm or irony or when linguistic information is incongruent with vocal affective cues [54, 55]. Findings from the eight studies reviewed demonstrate that TBI participants are not only impaired in recognizing emotions and affect through prosody but that they rely more heavily on linguistic cues alone in tasks requiring accuracy in decoding emotion and affect through auditory cues when the information delivered through prosody and semantics is incongruent. Adults without history of TBI mostly attend to prosodic (instead of linguistic) cues in these situations, a strategy that is particularly helpful in tasks requiring the detection of intentions such as irony and sarcasm [39, 56, 57]. Since studies on prosodic processing among TBI patients are scarce, it is unclear whether TBI patients attend to the linguistic rather than prosodic cues because they have difficulty processing prosody or because attentional impairments make it difficult to recognize the incongruence and lead to a less appropriate strategy for accuracy in decoding. Prosodic impairments were, however, still evident after controlling for cognitive or executive functions. Finally, both studies that used phonological processing tasks as control tasks for auditory processing deficits found that TBI patients presented with impairments in decoding prosody [33, 34].

Some may argue that impaired performance in recognizing emotion through prosody can be attributed to deficits in the brain’s ability to process emotions regardless of medium of delivery, rather than impairment in prosodic processing per se. Given that different sources of linguistic content, prosodic features, facial expression and body movements must be compared and integrated in some manner during interpersonal events, it is not surprising that cues presented in one modality (e.g. auditory) typically interact with cues presented in another modality (e.g. visual) [58]. However, although processing facial expressions (visual modality) and emotional speech prosody (auditory modality) can interact during information processing, they can also occur independently and may have individualized brain processing paths. For example, Adolphs and Tranel [59] found that while the human amygdala was particularly important for recognizing emotional meaning conveyed through facial expressions, but not prosody, the extra-amygdalar structures in the right hemisphere was particularly important for recognizing emotion conveyed through prosody, and not facial expressions.

Findings on effects produced by severity of GCS, age at injury, time-elapsed since injury and SES are mostly heterogeneous, which points to several implications for future research. First, future studies should be more consistent in collecting and reporting these variables, given that, as we observed during the course of this review, these factors may be related with both communicative and prosodic processing outcomes. Secondly, there is a need for longitudinal investigations assessing auditory and prosodic processing at shorter and consistent periods of time post-injury so that we can ascertain with more certainty if the outcomes observed can be linked with the assumed event that caused it and not confounds (repeated head or physical injuries). Just within the eight studies examined, time elapsed since injury ranged from immediately after injury to 25 years post-event, and age at injury, ranged from 8 to 70 years. Criteria for determining severity of TBI relies on differing scores and combinations of GCS, PTA, LOC and CT scans. Neuroplasticity and the brain's ability to re-learn in potentially adaptive circumstances may reduce the amount of prosodic impairments sustained post injury. Hence longitudinal examinations of prosodic impairments over long periods of time post injury would provide important information on prosodic rehabilitation and should be considered in future studies. Furthermore, it may be important to collect and report any prior music education or music training that participants may have received, as past research has shown that music training and exposure to music can improve prosodic processing and decoding of speech prosody, at least among healthy participants [22, 25, 26].

Studies included in our review found an association between temporal lobe damage, of both hemispheres and prosodic impairments. This is consistent with past neuroimaging research studies that have found that both processing of melodic pitch alterations [60] and processing of prosody of words [61] lead to activation of the anterior and posterior regions of the superior temporal sulci. As both rely on processing of prosodic stimuli and melodic contours instead of verbal or cognitive mechanisms, it is important to examine the prosodic processing aspects of prosodic impairments rather than focusing on the cognitive or verbal mechanisms. Future neuroimaging studies for TBI patients exhibiting prosodic impairments are much needed to identify specific neuro-structures and pathways damaged by TBI that could lead to prosodic processing impairments.

Finally, the association between communication deficits potentially caused by prosodic processing impairments experienced by TBI survivors and psychiatric, behavioural, social and employment challenges is not surprising. Past research has found that individuals with a history of TBI have higher odds of being diagnosed for depression, anxiety or both, ADHD, suicide ideation and attempt, hazardous drinking and unemployment or decreased productivity post-TBI [1, 12, 15, 17, 18, 21, 62]. These issues eventually lead to functional impairments and overall reduction in quality of life [63, 64]. Considering that prosodic processing impairments, communication deficits and emotion recognition deficits are also associated with psychosocial problems [53, 65], it raises the importance of identifying prosodic processing issues at early stages of the injury to reduce medical burden and improve outcomes for survivors of TBI.

### Limitations and future direction

Readers should be mindful of our study’s limitations. The small number of studies included in this review as well as the under-reporting and the heterogeneous pattern of patient characteristics and etiological variables observed between studies meant that many factors could not be analyzed and results between studies may not be comparable. Furthermore, most of the studies included examined prosodic impairment post-TBI despite the initial literature search terms that included a large and comprehensive list of language disorders. It is alarming that none of the studies reporting post-TBI aphasia have concurrently assessed for prosodic processing, despite prosodic processing deficits being common among aphasic patients [65–67]. This is especially important for patients who experience communication difficulties post-TBI despite scoring above cut-offs for typical standardized language tests, as the real issue may be prosodic processing impairments that are scarcely assessed in these tests. Finally, future studies in this area should also collect and analyze data regarding psycho-social and employment post-TBI, as they could be correlated with prosodic processing impairments as well as functional outcomes and recovery.

## Conclusions

This review highlights the importance of assessing TBI patients for prosodic processing impairments. TBI patients not only experience prosodic processing impairments that lead to difficulties in effective communication but such impairments often co-occurred with psychiatric issues, behavioural problems and reported unemployment post-TBI. Prosodic processing impairments appear to be correlated with TBI severity, location of brain lesion and time-elapsed since injury, though some of the correlations were inconsistent and warrant further examination.

It is important that future studies collect and report comprehensive details about severity of TBI, location of brain injury and time elapsed since injury, as they could influence the extent of prosodic processing impairments and recovery long term. If future studies could elucidate correlations between prosodic processing and TBI severity, prosodic processing screening could potentially be a valuable neuropsychological marker of TBI diagnosis and recovery. Prosodic processing is crucial for effective functional communication, and impairments in this domain could be a contributing factor to the many psychological problems and poor social outcomes observed in TBI patients. Treating prosodic processing issues in a timely fashion could result in significantly better functional outcome and help in costs reduction involved in the care for this population.

## Appendix 1

**Table 4:** MEDLINE search terms

	Search type	Actions
1	(traumatic brain injury or tbi).mp. [mp = title, abstract, original title, name of substance word, subject heading word, keyword heading word, protocol supplementary concept word, rare disease supplementary concept word, unique identifier]	24457
2	brain injur$.mp. or exp Brain Injuries/	70524
3	brain edema.mp. or "wounds and injuries"/[mp = title, abstract, original title, name of substance word, subject heading word, keyword heading word, protocol supplementary concept word, rare disease supplementary concept word, unique identifier]	78721
4	cerebral hemorrhage, traumatic.mp. or exp Cerebral Hemorrhage, Traumatic/or exp Craniocerebral Trauma/	127721
5	cerebrovascular trauma.mp. or exp Cerebrovascular Trauma/	5733
6	diffuse axonal injury.mp. or exp Diffuse Axonal Injury/or exp Head Injuries, Closed/	8599
7	diffuse axonal injuries.mp.	42
8	glasgow coma scale.mp. or exp Glasgow Coma Scale/	10497
9	glasgow outcome scale.mp. or exp Glasgow Outcome Scale/or exp Subarachnoid Hemorrhage/	19653
10	exp Post-Concussion Syndrome/or exp Brain Concussion/or concussion.mp.	6834
11	((head or crani* or cerebr* or brain* or forebrain* or skull* or hemispher* or intra?cran*) adj3 (injur* or trauma* or damag* or lesion* or wound* or oedema* or edema* or contusion* or concus* or fracture)).ti,ab.	146221
12	rancho los amigos.mp.	142
13	((unconscious* or coma* or concus* or 'persistent vegetative state') adj3 (injur* or trauma* or damag* or wound* or fracture* or contusion* or haematoma* or hematoma* or haemorrhag* or hemorrhag*)).ti,ab.	3368
14	(closed head injury or closed-head injury or CHI or closed-head injuries or closed head injuries).mp. [mp = title, abstract, original title, name of substance word, subject heading word, keyword heading word, protocol supplementary concept word, rare disease supplementary concept word, unique identifier]	129420
15	or/1-14	438225
16	aphasia.mp. or exp Aphasia, Wernicke/or exp Aphasia/or exp Aphasia, Primary Progressive/	14090
17	exp Language Disorders/or exp Speech Disorders/or prosodic impairment.mp. or exp Speech Perception/	57940
18	speech prosody.mp. or exp Speech Acoustics/	5883
19	(dysprosody or prosodic impairments).mp. [mp = title, abstract, original title, name of substance word, subject heading word, keyword heading word, protocol supplementary concept word, rare disease supplementary concept word, unique identifier]	68
20	((prosod* or speech* or language* or communicat* or linguist* or vocal*) adj3 (damag* or disorder* or difficult* impair* or challeng*)).ti,ab.	8582
21	((emotion* or pitch* or syllab* or phone* or affect* or voice* or vocal*) adj3 (recognition* or discrimination* or distinguish* or differentiat* or identif* or process*)).ti,ab.	44435
22	or/16-21	110875
23	15 and 22	5305
24	limit 23 to case reports	1236
25	23 not 24	4069
26	limit 25 to (letter or meta analysis)	36
27	25 not 26	4033
28	limit 27 to systematic reviews	55
29	27 not 28	3978
30	limit 29 to english language	3458
31	limit 30 to animals	149
32	30 not 31	3309
33	remove duplicates from 32	3261
34	limit 33 to yr = "1980 –Current”	2963

## Appendix 2

**Table 5:** STROBE checklist rating for each article

Article	WL: STROBE criteria met	WL: STROBE criteria not met	SW: STROBE criteria met	SW: STROBE criteria not met	Discrepancies
Dennis et al. [40]	1b), 2, 3, 4, 5, 6, 7, 8, 9, 10, 11, 12, 13a), 13b), 14, 15, 16, 17, 18, 19, 20, 21, 22	1a), 13c)	1b), 2, 3, 4, 5, 6, 7, 8, 9, 10, 11, 12, 13a), 13b), 14, 15, 16, 17, 18, 19, 20, 21, 22	1 a), 13c)	
Dimoska et al. [38]	1b), 2, 3, 4, 5, 6, 7, 8, 9, 10, 11, 12, 13a), 13b), 14, 15, 16, 17, 18, 19, 20, 21, 22	1a), 13c)	1b), 2, 3, 4, 5, 6, 7, 8, 9, 10, 11, 12, 13a), 13b), 14, 15, 16, 17, 18, 19, 20, 21, 22	1a), 13c)	
Ietswaart et al. [36]	1, 2, 3, 4, 5, 6, 7, 8, 9, 10, 11, 12, 13a), 13b), 14, 15, 16, 17, 18, 19, 20, 22	13c), 21	1, 2, 3, 4, 5, 6, 7, 8, 9, 10, 11, 12, 13a), 13b), 14, 15, 16, 17, 18, 19, 20, 21, 22	13c)	21 (consensus: criteria is not met)
McDonald and Saunders [39]	1b), 2, 3, 4, 5, 6, 7, 8, 9, 10, 11, 12, 13a), 13b), 14, 15, 16, 17, 18, 19, 20, 21	1a), 13c), 22	1b), 2, 3, 4, 5, 6, 7, 8, 9, 10, 11, 12, 13a), 13b), 14, 15, 16, 17, 18, 19, 20, 21	1a), 13c), 22	
Milders et al. [37]	1b), 2, 3, 4, 5, 6, 7, 8, 9, 10, 11, 12, 13a), 13b), 14, 15, 16, 17, 18, 19, 20, 21	1a), 13c), 22	1b), 2, 3, 4, 5, 6, 7, 8, 9, 10, 11, 12, 13a), 13b), 14, 15, 16, 17, 18, 19, 20, 21	1a), 13c), 22	
Schmidt et al. [33]	1, 2, 3, 4, 5, 6, 7, 8, 9, 10, 11, 12, 13a), 13b), 14, 15, 16, 17, 18, 19, 20, 21	13c), 22	1, 2, 3, 4, 5, 6, 7, 8, 9, 10, 11, 12, 13a), 13b), 14, 15, 16, 17, 18, 19, 20, 21	13c), 22	
Schmidt et al. [34]	1b), 2, 3, 4, 5, 6, 7, 8, 9, 10, 11, 12, 13a), 13b), 14, 15, 16, 17, 18, 19, 20, 21, 22	1a), 13c)	1b), 2, 3, 4, 5, 6, 7, 8, 9, 10, 11, 12, 13a), 13b), 14, 15, 16, 17, 18, 19, 20, 21, 22	1a), 13c)	
Zupan and Neumann [35]	1b), 2, 3, 4, 5, 6, 7, 8, 9, 10, 11, 12, 13a), 13b), 14, 15, 16, 17, 18, 19, 20, 21	1a), 13c), 22	1b), 2, 3, 4, 5, 6, 7, 8, 9, 10, 11, 12, 13a), 13b), 14, 15, 16, 17, 18, 19, 20, 21	1a), 13c), 22	

## Abbreviation

TBI: Traumatic brain injury
